# SLC15A2 genomic variation is associated with the extraordinary response of sorafenib treatment: whole-genome analysis in patients with hepatocellular carcinoma

**DOI:** 10.18632/oncotarget.3758

**Published:** 2015-04-22

**Authors:** Yeon-Su Lee, Bo Hyun Kim, Byung Chul Kim, Aesun Shin, Jin Sook Kim, Seung-Hyun Hong, Jung-Ah Hwang, Jung Ahn Lee, Seungyoon Nam, Sung Hoon Lee, Jong Bhak, Joong-Won Park

**Affiliations:** ^1^ Cancer Genomics Branch, National Cancer Center, Goyang, Republic of Korea; ^2^ Center for Liver Cancer, National Cancer Center, Goyang, Republic of Korea; ^3^ The Genomics Institute, Biomedical Engineering, UNIST, Ulsan, Republic of Korea; ^4^ Molecular Epidemiology Branch, National Cancer Center, Goyang, Republic of Korea; ^5^ Liver and Pancreatobiliary Cancer Branch, National Cancer Center, Goyang, Republic of Korea; ^6^ New Experimental Therapeutics Branch, National Cancer Center, Goyang, Republic of Korea; ^7^ Personal Genomics Institute, Genome Research Foundation, TheragenEtex, Suwon, Republic of Korea; ^8^ Theragen Bio Institute, TheragenEtex, Suwon, Republic of Korea; ^9^ Department of Preventive Medicine, Seoul National University College of Medicine, Seoul, Republic of Korea

**Keywords:** hepatocellular carcinoma, extraordinary response, sorafenib, whole-genome sequencing, genome variations

## Abstract

Reliable biomarkers are required to predict the response to sorafenib. We investigated genomic variations associated with responsiveness to sorafenib for patients with unresectable hepatocellular carcinoma (HCC). Blood samples from 2 extreme, 2 strong and 3 poor responders to sorafenib were subjected to whole-genome analysis. Then, we validated candidate genomic variations with another 174 HCC patients, and performed *in vitro* functional analysis and *in silico* analyses. Genomic data of >96 gigabases/sample was generated at average of ~34X sequencing depth. In total, 1813 genomic variations were matched to sorafenib responses in clinical data; 708 were located within regions for sorafenib-target genes or drug absorption, distribution, metabolism, and excretion (ADME)-related genes. From them, 36 variants were within the coding regions and 6 identified as non-synonymous single-nucleotide variants from 4 ADME-related genes (*ABCB1, FMO3, MUSK*, and *SLC15A2*). Validation genotyping confirmed sequencing results and revealed patients genotype for rs2257212 in *SLC15A2* showed longer progression-free survival (HR = 2.18). *In vitro* study displayed different response to sorafenib depending on the genotype of *SLC15A2*. Structural prediction analysis revealed changes of the phosphorylation levels in protein, potentially affecting sorafenib-associated enzymatic activity. Our finding using extreme responder seems to generate robust biomarker to predict the response of sorafenib treatment for HCC.

## INTRODUCTION

Hepatocellular carcinoma (HCC) is one of the most common types of cancers (with the highest prevalence in the Asia-Pacific region) and the third leading cause of cancer death worldwide. [1] Because this disease is mostly diagnosed at an advance stage, potentially curative therapies are effective in less than 30–40% of HCC patients. [2, 3] While systemic therapies are indicated for advanced HCC, no effective systemic therapy for patients with advanced HCC existed until the development of sorafenib therapy. Sorafenib is a multikinase inhibitor that targets HCC angiogenesis, and it has been shown to impart survival benefits (2.8 and 2.3 months, respectively) on patients in Western and Eastern phase III randomized controlled trials. [4, 5] These trials have established sorafenib as a first-line treatment option for patients with advanced HCC, who have sufficiently preserved liver function. [2, 6]

Sorafenib is known to inhibit receptor tyrosine kinases in cell membranes (e.g., vascular endothelial growth factor receptors and platelet-derived growth factor receptor-beta) and downstream intracellular serine/threonine kinases (e.g., Raf-1, wild-type B-Raf, and mutant B-Raf). These kinases are involved in tumor cell proliferation and tumor angiogenesis. In a previous study, sorafenib treatment resulted in dose-dependent inhibition of cell proliferation and induction of apoptosis in human HCC cells lines *in vitro* [7]; however, the precise underlying responsible mechanisms remain largely unknown.

Two phase III clinical trials have shown that about 50–60 percent of the study subjects are non-responders to sorafenib treatment, and reliable biomarkers are needed for predicting treatment responses prior to therapy initiation. [8, 9] In practice, identification of good responders to sorafenib treatment may be critical for personalized therapy for management of advanced HCC. Unfortunately, no such reliable biomarkers or clinical factors currently have been identified to date. In addition, understanding the reasons for such differential responses is essential as is identifying stratification factors such as tumor molecular profiles and individual genetic differences in drug metabolism.

To distinguish good responders to sorafenib treatment from the poor responders, it is necessary to identify genes or markers related to the sorafenib response. However, conventional technologies such as massive PCR amplification and Sanger sequencing are time consuming and expensive; therefore, a single laboratory cannot undertake such a study independently. Recently developed molecular genetics technologies such as next-generation sequencing (NGS) are innovative research tools for detecting genomic variations and are practical methods for detecting mutations with high accuracy. [10] In particular, whole-genome sequencing by NGS can be used to detect all genomic variations in exons, introns, and regulatory regions such as promoters and enhancers, [11, 12] allowing a systemic approach to biomarker identification. Consequently, challenges to identify genomic variations associated with drug response is emerged by analyzing the genome of exceptional responder by NGS. [13] As an effort to this, The National Cancer Institute (NCI) is funding for researches which are focused to “super responder” with hope to further identify patients of good responder using knowledge generated from super responder.

In this study, we present the results of a whole-genome sequencing analysis to identify sorafenib-response markers and genes using the peripheral blood of HCC patients treated with sorafenib. From the patients who were treated with sorafenib, we selected two extraordinary responders who showed time-to-progression (TTP) more than 38 months and two good responders. With further three poor responder who showed less than 5 month TTP, whole genome analysis was performed followed by bioinformatics analysis to compare their genomic variations. We identified genome-wide germline variations associated with the response to sorafenib treatment, especially in sorafenib-candidate targets and in absorption, distribution, metabolism, excretion (ADME) genes. Furthermore, we discovered that non-synonymous single-nucleotide variations (ns SNVs) in solute carrier family 15 (H+/peptide transporter) member 2 (SLC15A2) were functionally relevant to sorafenib metabolism by *in silico* and *in vitro* functional study. This finding was validated by genotyping of another 174 patients with HCC which revealed statistically significant progression-free survival (PFS) rate depending on genotype. This is the first report of precise identification of SNVs as associated with the extraordinary response to sorafenib treatment, and our results may prove useful for classifying patients with advanced HCC on the basis of outcomes from sorafenib treatment.

## RESULTS

### Patients demographics

Table 1a shows the baseline demographics and clinical characteristics of the seven enrolled patients which were analyzed by whole-genome sequencing. Five (71.4%) patients were male and HBsAg-positive. Their median age was 60 years, and all were Child-Pugh class A. The seven patients were at Barcelona Clinic of Liver Cancer (BCLC) stages B (*n* = 4) and C (*n* = 3). The median of maximum tumor size was 45 mm, and two patients exhibited macrovascular invasion. According to time-to-progression (TTP) with sorafenib treatment, patients were arbitrarily classified into as being good responders (*n* = 4, Nr. 1, 2, 3 and 4 in Table 1a) harboring two extraordinary responders (Nr. 1 and 2) and poor responders (*n* = 3; Nr.5, 6 and 7 in Table 1a). A validation cohort (Table 1b) comprised 174 patients, with a median age of 56 years (interquartile range [IQR], 49–63) and 84.5% were male. According to BCLC staging system, the number of patients with stage A, B, and C HCC were 2 (1.2%), 35 (20.1%), and 137 (78.7%), respectively. Twenty patients (11.5%) were administered sorafenib as an initial treatment. The median overall survival (OS) and PFS of the validation cohort was 11.0 months (95% CI, 8.9–12.7) and 4.4 months (95% CI, 3.7–5.0), respectively.

**Table 1a:** Baseline demographic and clinical characteristics of enrolled patients (*n* = 7)

Patient number	1	2	3	4	5	6	7
Sex	Female	Male	Male	Male	Female	Male	Male
Age	54	45	47	60	61	66	68
Etiology	HBV	HBV	HBV	HBV	HBV	HCV	HCV
mUICC stage	IVa	II	III	IVa	II	IVa	III
BCLC stage	C	B	B	C	B	C	B
Tumor morphology	Infiltrative	Multinodular	Multinodular	Multinodular	Multinodular	Multinodular	Multinodular
Macrovascular invasion	Yes	No	No	Yes	No	No	No
Maximum size of tumor (mm)	60	45	72	13	15	83	20
Child-Pugh class	A	A	A	A	A	A	A
Serum AFP (ng/mL)	3505.9	3104.7	9.6	14	730.6	123016	23.9
TTP (mo)	55.5	38.1	9.3	8.1	4.7	3.5	4.3
Follow-up (mo)	62.2	55.4	60.5	33.1	4.9	14.5	15.5
Sorafenib response	good	good	good	good	poor	poor	poor

mUICC, Modified International Union against Cancer; BCLC, the Barcelona Clinic of Liver Cancer; TTP, time to progression; HBV, hepatitis B virus; HCV, hepatitis C virus

**Table 1b:** Baseline characteristics of the validation cohort (*n* = 174)

Patient Characteristics (*n*, [IQR])		Tumor Characteristics	
Age (years)	56 [49–63]	Size of the largest nodule in the liver	
Male	147 (84.5%)	None	14 (8.1%)
Etiology		≤ 2 cm	22 (12.6%)
HBV	135 (77.6%)	2–5 cm	47 (27.0%)
HCV	13 (7.5%)	>5 cm	91 (52.3%)
HBV + HCV	2 (1.2%)		
Alcoholic	13 (7.5%)		
NBNCNA	11 (6.3%)		
		BCLC	
Cirrhosis	140 (80.5%)	Stage A/B/C	1.2%/20.1%/78.7%
Bilirubin (mg/dL)	1.1 [0.7–1.6]		
Prothrombin time (INR)	1.05 [0.9–1.15]	Massive/infiltrative type	87 (50.0%)
Creatinine (mg/dL)	1.1 [1.0–1.2]	Vascular invasion	95 (54.6%)
Albumin (g/dL)	3.9 [3.5–4.2]	Portal vein invasion	85 (48.9%)
AST (IU/L)	63.5 [43–106]	Hepatic vein invasion	25 (14.4%)
ALT (IU/L)	37 [26–72]	Extrahepatic metastasis	84 (48.3%)
Child-Pugh class (A/B)	82.8%/17.2%	AFP (ng/mL)	181.5 [16.3–2544.5]
MELD*	8.8 [7.7–10.6]	≥ 200	85 (48.9%)

Number (proportion) or median [interquartile range] are shown.

HBV, hepatitis B virus; HCV, hepatitis C virus; NBNCNA, non-B/non-C/non-alcoholic; INR, internationalized normalized ratio; MELD, Model for End-Stage Liver Disease; AFP, alpha-fetoprotein; UICC, International Union Against Cancer; BCLC, Barcelona Clinic Liver Cancer

* The MELD score was calculated according to the original formula without rounding or lower and upper bounds in the variables and the final score.

### Whole-genome analysis of patients genome by NGS

On average, >96 gigabase sequences per sample were generated with a ~32X mapping depth minimum and at a mapping rate of over 95% to the reference genome (NCBI build 37, HG19; Supplementary Table 1). This baseline value of whole-genome sequencing indicates that we obtained sufficient qualified sequencing reads to cover the entire genome. Using the final mapped bases, we constructed a genomic profile database for detecting SNVs, copy number variations, and structural variations. For each sample, we identified approximately 0.6 – 0.8 million small indels and approximately 3.9 – 4.1 million SNVs. The majority of these SNVs was located in the intron, but a large proportion of SNVs was found in coding regions and splicing sites—including missense, nonsense, or frameshift mutations—which may cause functional changes of affected genes (Supplementary Table 2). The number of functional SNVs was approximately the same for all samples without any significant difference between good and poor responder. All data generated by whole genome sequencing was deposited in public database (NCBI SRA, Accession Nr. SRS 428124). Chip data generated by the genome-wide Axiom array were used for quality control of the identified SNVs (>99% concordance rate for each samples; Supplementary Table 3).

### Identification of sorafenib response-associated variations

**Figure 1:**
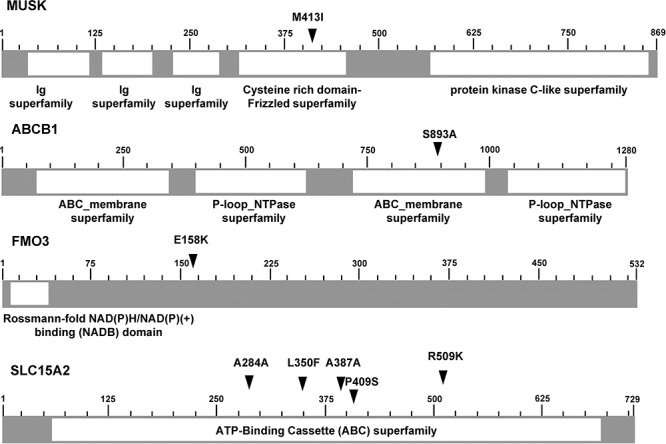
Schematics of six non-synonymous SNVs located in 4 genes Schematics of six non-synonymous SNVs located in four genes (MUSK, ABCB1, FMO3, and SLC15A2), illustrated with functional domains (arrows indicate the locations of variants; numbers indicate the position of amino acids.)

Genetic pattern analysis was used to identify genetic variations associated with drug responsiveness in the genomes of patients. Seven patients were assigned into two groups according to their sorafenib-responsiveness (good or poor responders). Alleles different from the human reference genome were coded as “A,” whereas alleles found the same as the reference genome were coded as “B.” This classification conferred four different genetic patterns in the response to sorafenib treatment, as shown in Supplementary Table 4. Subclasses 1 and 4 were dominant models in which “A” or “B” alleles dominantly contributed to the good sorafenib response, whereas subclasses 2 and 3 were recessive models whereby “A” or “B” homozygous alleles contributed to the good response to sorafenib treatment. We uncovered 1813 SNVs that were perfectly matched to one of these subclasses and identified as being 100% associated (Supplementary Table 5), 708 of which were located in the sorafenib target candidate genes or sorafenib ADME candidate genes (Supplementary Tables 6 and 7). Among the 708 SNVs, 36 variations were SNVs located in genomic regions, 15 of which were located in the coding regions of nine genes (Table 2), all confirmed as being true-positive variants by Sanger sequencing. Thirteen of these 15 coding SNVs were confirmed to be located in the drug responsiveness-related genes, whereas two of them were candidates as sorafenib target gene. Six coding SNVs were non-synonymous, which cause amino-acid change and possibly damage the functions of their encoded proteins, and located in four genes: MUSK was a sorafenib target gene candidate; ABCB1, FMO3, and SLC15A2 were ADME genes (Fig. 1).

**Table 2:** Coding variants in candidate genes

Gene	Chromosome^a^	Position^b^	Ref^c^	Variant^d^	Ref amino acid^e^	Variant amino acid^f^
ABCB1	chr7	87160618	A	C	S	A
ALDH3B1	chr11	67795299	G	A	P	P
ALDH3B1	chr11	67795353	G	A	L	L
CYP21A2	chr6	32006317	C	T	L	L
DDR1	chr6	30865204	A	C	P	P
FMO3	chr1	171076966	G	A	E	K
MUSK	chr9	113538122	G	A	M	I
SLC15A2	chr3	121646641	A	G	A	A
SLC15A2	chr3	121643804	C	T	L	F
SLC15A2	chr3	121641693	G	A	A	A
SLC15A2	chr3	121647286	C	T	P	S
SLC15A2	chr3	121648168	G	A	R	K
SLC22A15	chr1	116534852	C	T	S	S
SLC7A7	chr14	23282449	C	T	S	S
SLC7A7	chr14	23282110	A	G	I	I

a Chromosome on which the variation was located

b Nucleotide position of the variant allele in the human reference genome sequence version 19/build 37

c Nucleotide at the same position in human reference genome sequence version 19/build 37

d Nucleotide at the variation site

e Amino acid encoded by the corresponding codon in the reference sequence

f Amino acid encoded by the corresponding codon in the variant sequence

### Pathway analysis

To further investigate sorafenib-associated variations at the pathway level, all variations were analyzed by gene positioning in the functionally annotated KEGG database. The genes that harbored 1813 genomic variations were mapped in the KEGG pathways; significantly affected pathways (*P* < 0.001) are displayed in Supplementary Table 8. Interestingly, the outcome of the pathway analysis was in accordance with that of the marker analysis, and the drug metabolism pathway (including its components such as FMO3 and ALDH3B1) were found to be the most significantly affected. This suggests that these genetic differences in the patients with HCC are involved in mechanisms implicated in different responses to sorafenib treatment.

### Validation of sorafenib-associated SNVs in SLC15A2 and functional analysis

**Figure 2:**
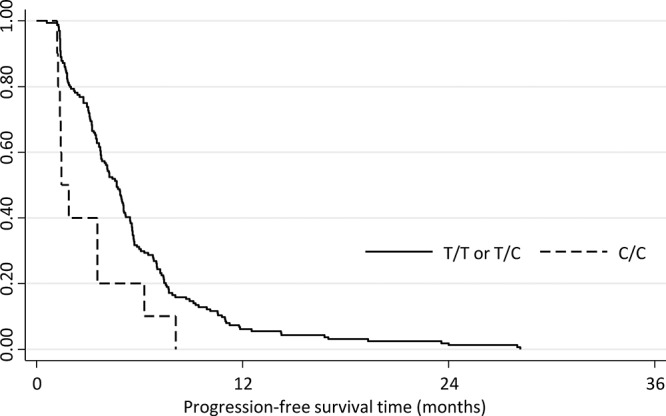
Kaplan-Meier plot depicting the progression-free survival of the validation cohort according to the SLC15A2 genotype Progression-free survival was significantly longer for patients with C/C genotype compared with those with C/T or T/T genotypes (HR: 2.18; 95% CI, 1.15–4.15; *P* = 0.018).

Since SLC15A2 belongs to the solute carrier family that supposedly participates in drug transport, we wanted to validate the genomic variations in SLC15A2. From five coding variants in SLC15A2 identified by NGS analysis, we selected three non-synonymous SNVs (L350F, P409S, R509K), which may cause functional alteration in the gene product, and analyzed the genotypes for an additional 174 HCC patients (Table 1b). We obtained individual genotypes of these three SNPs, which displayed complete linkage disequilibrium (Supplementary Table 9). Because of missing clinical data, we could only analyze PFS but not TTP. However, upon analyzing the association between genotypes and the PFS of these patients, we observed that cohorts of patients with C/C genotype for rs2257212 (correspond to L350F) showed shorter progression-free survival time compared to the group of patients with C/T or T/T genotype (HR, 2.18; 95% CI, 1.15–4.15; *P* = 0.018) as it was indicated by NGS analysis. The Kaplan-Meier plot displayed significant genotype-dependent difference for PFS of 174 patients (Fig. 2) which may be caused by different drug responsiveness for sorafenib. Additional analysis by pair-wise addition of SNPs in coding variants to Cox proportional hazard model with SLC15A2 did not significantly improve model predictability (data not shown).

**Figure 3:**
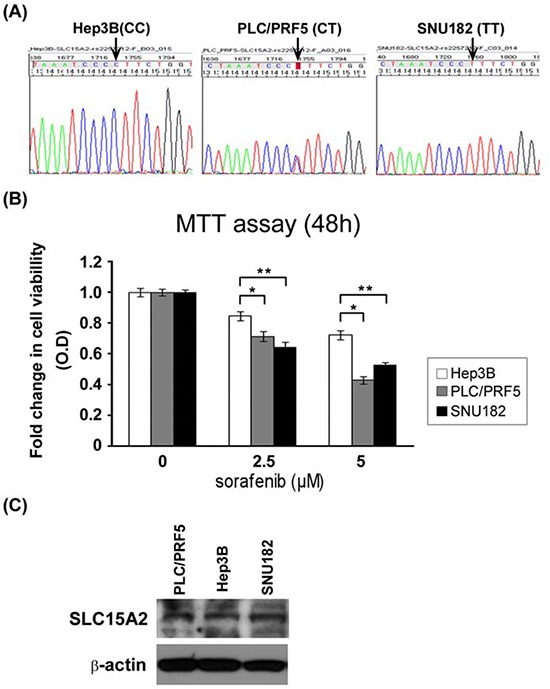
Functional analysis of SLC15A2 variant in liver cancer cell lines Three liver cancer cell lines were selected after their genotypes were validated by Sanger sequencing **A.** MTT assay after sorafenib treatment revealed that cell viability of the Hep3B cell line harboring the C/C genotype was considerably less affected than that of the SNU182 cell line harboring T/T genotype **B.** Western blot analysis showed similar expression SLC15A2 levels for all cell lines, irrespective of the genotype, which may indicate structural changes in the protein and which may be responsible for the differential drug response **C.** (** indicate *P* < 0.05, *** indicate *P* < 0.01)

Further, we analyzed the functional effect of a non-synonymous variant in SLC15A2. For *in vitro* functional analysis, we first identified the variant genotypes of rs2257212(L350F) in several human HCC cell lines and found that Hep3B, PLC/PRF5, and SNU182 possessed the C/C, C/T and T/T genotypes, respectively (Fig. 3A). Consistent with the results of whole-genome analysis and validation study, the T variant of rs2257212 in SLC15A2 showed a better response to sorafenib than the C variant. Although the proliferation of cell lines was inhibited by sorafenib treatment in a dose-dependent manner, SNU182 cells that harbored the T/T genotype displayed a better response to sorafenib than the Hep3B cells that harbored the C/C genotype (Fig. 3B). Additional western blot analysis showed similar SLC15A2 expression levels in all three cell lines (Fig. 3C), suggesting that structural, rather than expressional, changes were the cause of the function aberration.

## DISCUSSION

Cancer biomarkers predicting outcomes of drug treatment may be used to assess the probability that a patient will benefit from a particular regimen of cancer treatment. [14] Previous biomarker response studies have evaluated the correlation between plasma angiogenesis biomarkers, alpha-fetoprotein (AFP) level or vascular permeability-perfusion of dynamic magnetic resonance imaging, and responses to sorafenib in patients with advanced HCC. [15–17] However, finding reliable biomarkers is not an easy task and none of biomarkers are clinically applicable to date. Recent approaches focusing comprehensive genome analysis of extraordinary response patients give rise to hope to identify genomic alterations associate with drug response. Especially, two studies identified genomics alterations related to drug responses by analyzing “outlier” responder in lung cancer patients. [18, 19] Here, we analyzed four good responder of sorafenib and one of them is extreme outlier with more than 55 months TTP although disease stage at the beginning of treatment was IVa by mUICC stage. One other patient also showed extraordinary response to treatment with more than 38 months TTP. Our study represents the first effort to identify sorafenib-response variations and related genes through whole-genome analysis of HCC patients.

This study was conducted in a phase II clinical trial setting (named COTSUN), whereby the safety and efficacy of concurrent TACE and sorafenib in patients with HCC was evaluated. [20] TACE used as a locoregional therapy may not directly affect the sorafenib response and, in the present study, clinical characteristics were well collected and balanced between the good and poor responder cohorts; hence, we used blood samples from prospectively collected specimens from the COTSUN trial. Using blood samples from such a study is more convenient than using tumor specimens in terms of availability of biomarkers.

A total of 1813 genomic variations with 100% association for sorafenib responsiveness was discovered in this study. Among these, six non-synonymous SNVs were located in one sorafenib target gene (MUSK) and three ADME genes (ABCB1, FMO3, and SLC15A2), which could be considered as the member of main causes for sorafenib drug responsiveness, possibly by influencing protein function. To the best of our knowledge, this is the first to report suggesting that MUSK, ABCB1, FMO3, and SLC15A2 could be indicative for the response to sorafenib in patients with HCC. Among the four candidate genes that we identified in this study, ABCB1 is a member of the superfamily of ATP-binding cassette (ABC) transporters. Interestingly, for Asian nasopharyngeal carcinoma patients, polymorphisms in the ABCG2 and ABCB1 genes could be predictive markers of drug (irinotecan) activity. [21] The FMO3 gene is a type of flavin-containing monooxygenase, which is a member of an important class of drug-metabolizing enzymes. [22–24] Unfortunetly, the results from our validation set of patients and *in vitro* study did not confirm a meaningful association between these genes (ABCB1, FMO3) and the response to sorafenib (data not shown). These may be caused by marginal number of patients or multiple variations could be associated simultaneously which should be further analyzed.

The most interesting result of this study was the identification of non-synonymous single nucleotide variations in SLC15A2 as an important predictor of the response to sorafenib treatment in patients with HCC. SLC15A2 belongs to the family of proton-coupled peptide transporters that is responsible for the absorption of small peptides, as well as beta-lactam antibiotics and other drugs. [25] In our whole-genome analysis, the variations in this gene were found to be associated with sorafenib responsiveness and this result was validated in the practice-based cohort of 174 patients who were treated with sorafenib (Fig. 2; Table 1b). Patients with the T/T or C/T genotype displayed a significantly longer PFS than did patients with C/C genotypes (*P* = 0.003) maybe caused by better response to sorafenib. In the *in vitro* study, the suppression of HCC cell proliferation by sorafenib displayed greater dominance in SNU182 (an HCC cell line that was T/T homozygous for SLC15A2) compared to that in Hep3B (an HCC cell line that was C/C homozygous for SLC15A2). Taken together, our findings indicate that the functional impact of coding variants in SLC15A2 may affect sorafenib metabolism and responsiveness.

When we consider the results of *in vitro* assays in which similar SLC15A2 expression levels were observed in cell lines with different genotypes, the functional mechanism of the SLC15A2 variants should be related not to the change of expression level but to the structural changes caused by these variants. Although *in silico* analysis for three-dimensional structure prediction did not display substantial differences between both genotypes (data not shown), computational analysis (pubmed id: 15980458, kinasephos.mbc.nctu.edu.tw) of post-translational modifications revealed that protein generated from genes with variant genotypes (identified in good responders) may have more potential phosphorylation sites than wild type (identified in poor responders), as shown in Supplementary Table 10. The increased number of phosphorylation sites of SLC15A2 may delay the dephosphorylation of the protein, contributing to the longer activation required for SLC15A2, such that it could participate more in the mechanism of the sorafenib reaction. Together, patients with variant types of SLC15A2 may possess advantages in the effectiveness and responsive to sorafenib treatment because of the increased stability of the SLC15A2 protein which should be involved in the transport of this drug.

The analysis of gene-to-gene connection using the Liver hepatocellular carcinoma data in c-BioPortal (http://www.cbioportal.org/public-portal/) showed the association of SLC15A2 with cystic fibrosis transmembrane conductance regulator (CFTR) via PDZ domain containing 1 or 3 (PDZK1 or PDZD3; Supplementary Fig. 1). CFTR, a well-established member of the ATP-binding cassette (ABC) transporter superfamily, transport various molecules across extracellular and intracellular membranes. Polymorphisms of CFTR are known to be associated with benefit for clinical evaluation. [26, 27] Also, molecular alteration of PDZK1 may be associated with metabolic syndrome and drug-resistance phenotype in multiple myeloma. [28, 29] Additionally, many members of solute carrier family were revealed, especially the organic cation transporter OCT/OCT novel (OCTN) family. SLC22A4 (OCTN1) and SLC22A5 (OCTN2) were linked with SLC15A2 via PDZK1 and/or PDZD3. Interestingly, recent study reported that the polymorphisms of these genes are associated with prolonged TTP in unresectable gastrointestinal stromal tumors treated with imatinib therapy. [30] Taken together, the results of this network analysis provide additional evidence for the functional relevance of SLC15A2 in the response to sorafenib treatment.

SLC15A2 may transport many clinical and experimental therapeutic compounds, including beta-lactams, angiotensin-converting enzyme inhibitors, 5-aminolevulinic acid, bestatin, and pro-drugs such as Val-acyclovir and Val-ganciclovir. [31, 32] Previous reports have shown that different haplotypes of these variants exhibited markedly different *K*m values for glycyl-sarcosine (Gly-Sar). [33] Moreover, these variants revealed ethnic disparities for which Caucasian (0.371–0.500) and European (0.465–0.492) possessed lower frequencies of the T allele in than the Asian population did (0.744–0.800) [http://www.ncbi.nlm.nih.gov/projects/SNP/]. These disparate frequencies may provide one possible explanation for the differential responses to sorafenib treatment vis à vis ethnicity. Although validation with the independent cohort of this study displayed a predictive significance to SLC15A2 genetic variation, further prospective clinical studies are needed to confirm the predictive and/or prognostic values of SLC15A2 genetic variation in responses to sorafenib treatment.

In conclusion, we discovered genomic variants and their genes that should be associated with sorafenib responses for patients with HCC which could be used as biomarkers for predicting the effectiveness of drug responses. This is also the first report showing a significant influence of the SLC15A2 genotype on the response to sorafenib in patients with HCC; our results suggest that SLC15A2 is a novel gene involved in sorafenib metabolism. Although further prospective validation and functional studies are needed, our results provide the initial clues for classifying patients with HCC by predicting the outcome of sorafenib treatment and for tailoring individualized therapy for these patients.

## MATERIALS AND METHODS

### Patients and specimen collection

The whole genome of seven patients was used for NGS analysis. Six patients were selected from a cohort of 50 patients with HCC, who were enrolled in a phase II study of concurrent transarterial chemoembolization (TACE) and sorafenib treatment. [20] Another patient was selected from a patient cohort displaying a long-term (>48 weeks) response to sorafenib treatment. [34] HCC was diagnosed by histological examination or on the basis of clinicoradiologic criteria based on the guidelines of the Korean Liver Cancer Study Group and the National Cancer Center (NCC), Republic of Korea. [35] A validation cohort was derived from 408 patients with HCC who had been treated with sorafenib for greater than a 6-week period between June 2007 and March 2012 at the NCC. [36] Fifty-seven patients who were lost to follow up or who discontinued sorafenib treatment due to non-medical issues were excluded. Out of the 351 patients, blood samples were obtained from 174 patients and were subsequently used for genotyping (Supplementary Fig. 2). This study was conducted in accordance with the Declaration of Helsinki and International Conference on Harmonization-Good Clinical Practice. The study protocol was approved by the Institutional Review Board at the National Cancer Center (Goyang, Republic of Korea). All patients provided us with their written informed consent to undertake the study.

### Nucleic acid preparation and whole-genome sequencing

Genomic DNA was extracted from patient leukocytes by a MagAttract DNA Blood Midi Kit (Qiagen, Inc. Valencia, CA, USA) according to the manufacturer's protocol. The DNA quality was assessed using a Nanodrop spectrometer (Nanodrop Technologies, Wilmington, DE, USA). An absorbance 260/280 value greater than 1.7 was accepted for further analysis. Five micrograms of genomic DNA was sheared using a Covaris S series ultrasonicator (Covaris, Woburn, MA, USA). The fragments of sheared DNA were then end-repaired, A-tailed, ligated to pair-end adapters (Pair End Library Preparation Kit, Illumina, CA, USA), and amplified by PCR according to the manufacturer's protocol. The quality of the library and DNA concentration were determined using an Agilent 2100 BioAnalyzer (Agilent, Santa Clara, CA, USA) and quantified using a SYBR green qPCR protocol on a LightCycler 480 (Roche, Indianapolis, IN, USA) according to Illumina's library quantification protocol. Paired-end sequencing (2 × 100 bp) was performed on an Illumina HiSeq 2000 by using HiSeq Sequencing kits

### Read alignment and variation detection

Next, 90-bp paired-end sequence reads with ~300-bp inserts were aligned to the hg19 human reference genome (NCBI build 37) by using a BWA algorithm1 ver. 0.5.9. Two mismatches were permitted in the 45-bp seed sequence. SAMtools were used to remove PCR duplicates of the sequence reads, which may have been generated during the library construction process. The aligned reads were realigned at putative insertion/deletion (indel) positions by using the GATK IndelRealigner algorithm to enhance mapping quality. [37] Base quality scores were recalibrated using the GATK TableRecalibration algorithm.

### SNP and small indel analysis

Putative SNVs were called and filtered using the UnifiedGenotyper and VariantFiltration commands in GATK. The options used for SNP calling were a minimum of 5 to a maximum of 200 reads in terms of mapping depth with a consensus quality of 20, and the prior likelihood for heterozygosity value of 0.001. To obtain small indels, the dINDEL mode of the GATK UnifiedGenotyper was used.

### Validation of SNVs by using genome-wide SNP chips and Sanger sequencing

SNP genotyping to confirm the NGS data was performed using an Axiom genotyping solution with an Axiom Genome-Wide ASI 1 Array Plate (Affymetrix, Santa Clara, CA, USA) and a reagent kit according to the manufacturer's protocol. Total genomic DNA (200 ng) was used and the resulting data in the form of a DAT file was automatically saved as a CEL file. The CEL intensity file was normalized, and genotype calling was performed using Genotyping Console 4.1 (Affymetrix) with Axiom GT1 algorithms according to the manufacturer's instructions. The cutoff value for data quality control was a DISHQC ≥ 0.82 for hybridization and a QC call rate ≥ 97%.

Twenty-six SNVs, including 15 coding variations, were validated by conventional Sanger sequencing using dye-terminator chemistry. The sequences were analyzed using an automatic sequencer ABI 3730 (Applied Biosystems, Carlsbad, CA, USA). The target regions were amplified by PCR followed by direct sequencing or were cloned into TA vectors. Details of the PCR and sequencing primers are provided in Supplementary Table 11.

### Annotation and in silico analysis of variations

Each SNV was mapped onto the UCSC gene table according to its genomic features such as coding region, untranslated region (UTR), and introns. Non-synonymous SNV information was extracted by comparing UCSC (http://genome.ucsc.edu/) reference gene information. Pathway analysis was performed for the genes annotated to harbor significant mutations by using the Kyoto Encyclopedia of Genes and Genomes (KEGG; 32, http://www.genome.jp/kegg/) and Biocarta (http://www.biocarta.com/). For structural analyses of variations in SLC15A2, RefSeq NM_001145998 was used. Three-dimensional structures were generated using Phyre 2.0 (http://www.sbg.bio.ic.ac.uk/phyre2/html/page.cgi?id=index). Post-translational modification was analyzed using KinasePhos (kinasephos.mbc.nctu.edu.tw). Integrative analysis for SLC15A2 with publicly available data and pathways was performed by the cBioPortal website (www.cbioportal.org).

### Validation of candidate SNPs and Statistical analyses

To validate the candidate SNPs, 174 patients with HCC who had been treated with sorafenib were genotyped using the MassARRAY system (Sequenom, San Diego, CA, USA) as previously described.[38] Progression-free survival (PFS) was assessed by the Kaplan-Meier method, and the association between generic polymorphisms and risk for progression was assessed by Cox proportional hazard model with adjustment by stage of hepatocellular carcinomas. Hazard ratios and their 95% confidence intervals (95% CI) were computed. Data analyses were performed using STATA version 12.0 (Stata Corp, College Station, TX, USA)

### Validation and functional analysis of SLC15A2

Hep3B, PLC/PRF5 and SNU182 cells derived from human HCCs were obtained from the Korean Cell Line Bank (KCLB; Seoul, Republic of Korea) and cultured in RPMI-1640 (Invitrogen, Carlsbad, CA, USA) with 10% (v/v) FBS and 100 U/mL penicillin-streptomycin at 37°C in 5% CO_2_. Genomic DNA was extracted from cells using a MagAttract DNA mini M48 kit (Qiagen) according to the manufacturer's instructions. For analysis of the SLC15A2 genotypes, genomic DNA was amplified by PCR using SLC15A2 primers, forward: (5′-GGGTCTTGGGTGTAAATGGA-3′), reverse: (5′-CACACTTGGAGACCAGACGA-3′), and sequenced as described above. For the MTT assay, cells were plated in 96-well plates at a density of 1 × 10^4^ cells/well and treated with sorafenib for 48 h in RPMI-1640 medium. Next, the number of viable cells was measured by performing MTT assay (Promega Fitchburg, WI, USA) according to the manufacturer's instructions. For western blot analysis, 30 μg of each cell lysates was resolved on 4–12% NuPage gel (Invitrogen) and then transferred onto Immobilon (Millipore, Billerica, MA, USA). Immunoblotting was performed using an anti-SLC15A2 primary antibody (Santa Cruz Biotechnology, Santa Cruz, CA, USA) and anti-β-actin (Abcam, Cambridge, MA, USA). Protein bands were detected using WestZol (iNtRon, Gyeonggi, Republic of Korea).

## SUPPLEMENTARY FIGURES AND TABLES




