# Supplementary material for: SLC15A2 genomic variation is associated with the extraordinary response of sorafenib treatment: whole-genome analysis in patients with hepatocellular carcinoma
Source: Oncotarget. 2015 Apr 22;6(18):16449–60. doi: 10.18632/oncotarget.3758 (PMC4599281; doi:10.18632/oncotarget.3758)
Supplement: Supplementary file 1 [file oncotarget-06-16449-s001.pdf]

SUPPLEMENTARY FIGURES AND TABLES

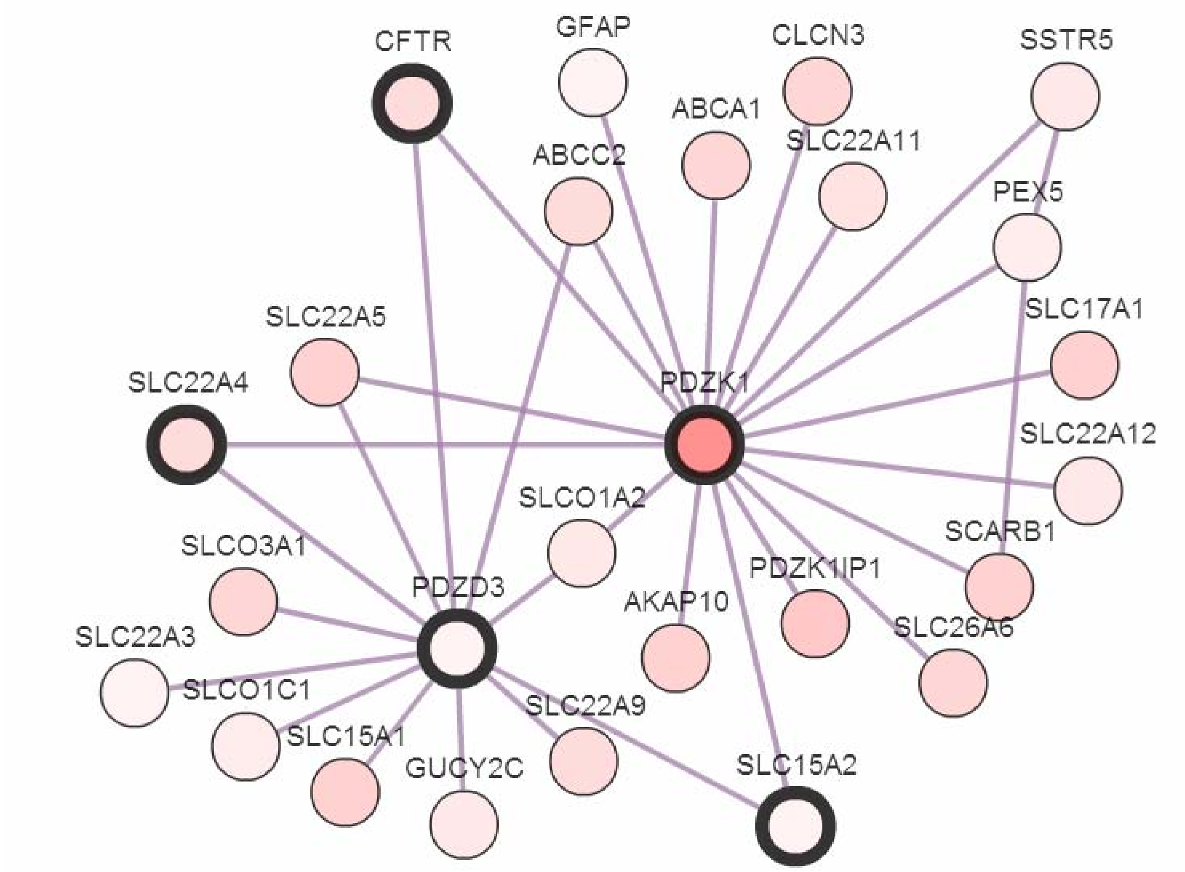

Supplementary Figure S1: Network analysis of SLC15A2. The network analysis using cBioPortal (www.cbioportal.org) revealed the association of SLC15A2 with CFTR via PDZK1 and PDZD3.

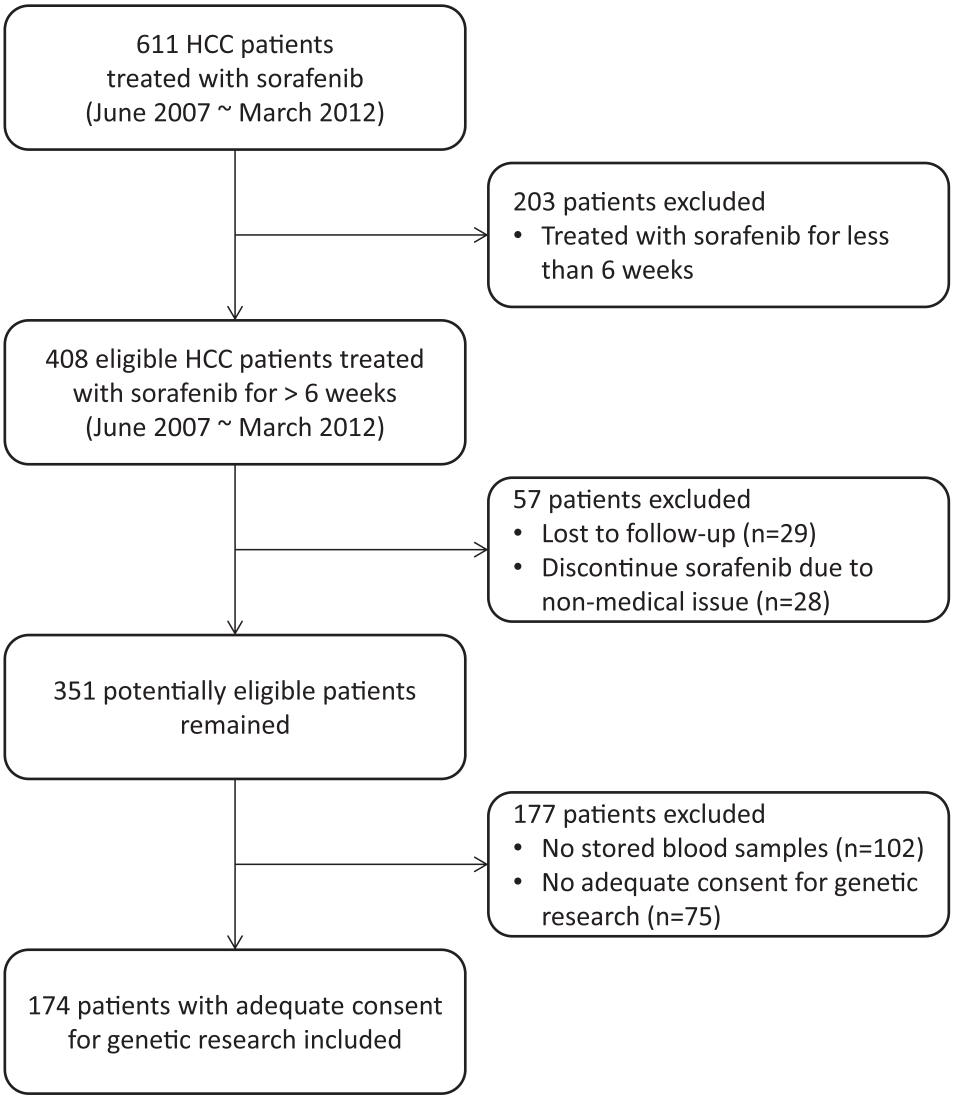

Supplementary Figure S2: Flow diagram of study subjects.

Supplementary Table S1. Sequencing yield and mapping depth for the patients genome

Patient number	Total bases (Gb)	Sequencing depth (fold)	Mapped bases (Gb)	Mapping depth (fold)	Mapping rate (%)	Single nucleotide variations	Small insertion/ deletion
1	183.07	63.98	169.49	59.23	92.58	4, 151, 040	818, 141
2	97.68	34.14	92.54	32.34	94.73	4, 005, 515	695, 051
3	197.83	69.14	182.18	63.67	92.09	4, 121, 239	824, 987
4	102.64	35.87	97.53	34.09	95.02	3, 990, 020	705, 627
5	99.39	34.74	94.73	33.11	95.32	4, 014, 993	701, 416
6	205.99	71.99	189.76	66.32	92.12	4, 139, 398	817, 540
7	111.23	38.87	105.76	36.96	95.08	4, 004, 111	718, 519

Supplementary Table S2. Variations in individual patient's genome

Patient number	cds[a]	splicing	intron	promoter	missense nsSNV[b]	Nonsense nsSNV[c]	frameshift
1	24, 594	119	1, 767,084	31, 181	11586	151	305
2	23, 661	105	1, 676,462	29, 239	11033	103	229
3	24, 555	113	1, 762,603	31, 025	11575	141	290
4	23, 414	104	1, 675,760	28, 428	10926	124	223
5	22, 892	95	1, 681,834	28, 939	10632	112	210
6	24, 523	113	1, 770,318	31, 030	11583	142	283
7	23, 322	114	1, 688,035	28, 826	10883	106	231

[a]cds, coding DNA sequence;
[b]missense nsSNV, non-synonymous single nucleotide variation, which results in a different amino acid
[c]Nonsense nsSNV, non-synonymous single nucleotide variation, which results in a premature stop codon

Supplementary Table S3. Concordance between SNVs and chip-based genotyping

Patient number	Concordant SNVs	Disconcordant SNVs	Concordance (%)
1	181, 424	240	99.87
2	174, 645	1, 489	99.15
3	178, 347	381	99.79
4	178, 194	302	99.83
5	181, 148	254	99.86
6	178, 842	288	99.84
7	177, 120	338	99.81

Supplementary Table S4. Possible genotypic classes dependent on Sorafenib response

Class	Good responder	Poor responder
Subclass1	AA, AB	BB
Subclass2	AA	AB, BB
Subclass3	BB	AA, AB
Subclass4	BB, AB	AA

A clinically significant improvement in time-to-progress more than 5 months was defined as good responders, while time-to-progress less than 5 months was defined as poor responders. This patient classification were further divided into 4 subclasses according to their genotypes. Different alleles from the human reference genome are coded as "A", while same alleles as the reference genome are coded as "B".

Supplementary Table S5. Number of SNVs in each subclasses

Class	Total	Candidates	Frequency of candidates
Subclass1	544	36	7%
Subclass2	731	66	9%
Subclass3	243	27	11%
Subclass4	295	9	3%

Supplementary Table S6. Sorafenib target candidates

Gene name	Nucleotide accession number	Protein accession number	Official full name
AATK	NM_004920	NP_004911	apoptosis-associated tyrosine kinase
ABL1	NM_005157	NP_005148	c-abl oncogene 1, receptor tyrosine kinase
ALK	NM_004304	NP_004295	anaplastic lymphoma receptor tyrosine kinase
ARAF	NM_001654	NP_001645	v-raf murine sarcoma 3611 viral oncogene homolog
BLK	NM_001715	NP_001706	B lymphoid tyrosine kinase
BMX	NM_001721	NP_001712	BMX non-receptor tyrosine kinase
BRAF	NM_004333	NP_004324	v-raf murine sarcoma viral oncogene homolog B1
BTK	NM_000061	NP_000052	Bruton agammaglobulinemia tyrosine kinase
CSK	NM_004383	NP_004374	c-src tyrosine kinase
DDR1	NM_001954	NP_001945	discoidin domain receptor tyrosine kinase 1
DDR2	NM_006182	NP_006173	discoidin domain receptor tyrosine kinase 2
DOK1	NM_001381	NP_001372	docking protein 1, 62kDa (downstream of tyrosine kinase 1)
EGFR	NM_005228	NP_005219	epidermal growth factor receptor (erythroblastic leukemia viral (v-erb-b) oncogene homolog, avian)
FER	NM_005246	NP_005237	fer (fps/fes related) tyrosine kinase
FGFR1	NM_010206	NP_034336	fibroblast growth factor receptor 1
FLT1	NM_002019	NP_002010	fms-related tyrosine kinase 1 (vascular endothelial growth factor/vascular permeability factor receptor)
FLT3	NM_004119	NP_004110	fms-related tyrosine kinase 3
FLT3LG	NM_001459	NP_001450	fms-related tyrosine kinase 3 ligand
FLT4	NM_002020	NP_002011	fms-related tyrosine kinase 4
HGF	NM_000601	NP_000592	hepatocyte growth factor (hepapoietin A; scatter factor)
HGS	NM_004712	NP_004703	hepatocyte growth factor-regulated tyrosine kinase substrate
IBTK	NM_015525	NP_056340	inhibitor of Bruton agammaglobulinemia tyrosine kinase
KDR	NM_002253	NP_002244	kinase insert domain receptor (a type III receptor tyrosine kinase)
LCK	NM_005356	NP_005347	lymphocyte-specific protein tyrosine kinase

(*Continued*)

Gene name	Nucleotide accession number	Protein accession number	Official full name
LMTK2	NM_014916	NP_055731	lemur tyrosine kinase 2
LMTK3	NM_001005511	NP_001005511	lemur tyrosine kinase 3
LTK	NM_002344	NP_002335	leukocyte receptor tyrosine kinase
MAPK1	NM_002745	NP_002736	mitogen-activated protein kinase 1
MATK	NM_002378	NP_002369	megakaryocyte-associated tyrosine kinase
MERTK	NM_006343	NP_006334	c-mer proto-oncogene tyrosine kinase
MET	NM_000245	NP_000236	met proto-oncogene (hepatocyte growth factor receptor)
MST1R	NM_002447	NP_002438	macrophage stimulating 1 receptor (c-met-related tyrosine kinase)
MUSK	NM_005592	NP_005583	muscle, skeletal, receptor tyrosine kinase
NTRK1	NM_002529	NP_002520	neurotrophic tyrosine kinase, receptor, type 1
NTRK2	NM_006180	NP_006171	neurotrophic tyrosine kinase, receptor, type 2
NTRK3	NM_002530	NP_002521	neurotrophic tyrosine kinase, receptor, type 3
PDGFRA	NM_006206	NP_006197	platelet-derived growth factor receptor, alpha polypeptide
PDGFRB	NM_002609	NP_002600	platelet-derived growth factor receptor, beta polypeptide
PDGFRL	NM_006207	NP_006198	platelet-derived growth factor receptor-like
PTK2	NM_005607	NP_005598	PTK2 protein tyrosine kinase 2
PTK2B	NM_004103	NP_004094	PTK2B protein tyrosine kinase 2 beta
PTK6	NM_005975	NP_005966	PTK6 protein tyrosine kinase 6
PTK7	NM_002821	NP_002812	PTK7 protein tyrosine kinase 7
RAF1	NM_002880	NP_002871	v-raf-1 murine leukemia viral oncogene homolog 1
RET	NM_009050	NP_033076	ret proto-oncogene
ROR1	NM_005012	NP_005003	receptor tyrosine kinase-like orphan receptor 1
ROR2	NM_004560	NP_004551	receptor tyrosine kinase-like orphan receptor 2
ROS1	NM_002944	NP_002935	c-ros oncogene 1 , receptor tyrosine kinase
RYK	NM_002958	NP_002949	RYK receptor-like tyrosine kinase
STYK1	NM_018423	NP_060893	serine/threonine/tyrosine kinase 1

(*Continued*)

Gene name	Nucleotide accession number	Protein accession number	Official full name
SYK	NM_003177	NP_003168	spleen tyrosine kinase
TEC	NM_003215	NP_003206	tec protein tyrosine kinase
TEK	NM_000459	NP_000450	TEK tyrosine kinase, endothelial
TIE1	NM_005424	NP_005415	tyrosine kinase with immunoglobulin-like and EGF-like domains 1
TNK1	NM_003985	NP_003976	tyrosine kinase, non-receptor, 1
TNK2	NM_005781	NP_005772	tyrosine kinase, non-receptor, 2
TXK	NM_003328	NP_003319	TXK tyrosine kinase
TYK2	NM_003331	NP_003322	tyrosine kinase 2
TYRO3	NM_006293	NP_006284	TYRO3 protein tyrosine kinase
TYROBP	NM_003332	NP_003323	TYRO protein tyrosine kinase binding protein
WNT1	NM_005430	NP_005421	wingless-type MMTV integration site family, member 1
WNT10A	NM_025216	NP_079492	wingless-type MMTV integration site family, member 10A
WNT10B	NM_003394	NP_003385	wingless-type MMTV integration site family, member 10B
WNT11	NM_004626	NP_004617	wingless-type MMTV integration site family, member 11
WNT16	NM_016087	NP_057171	wingless-type MMTV integration site family, member 16
WNT2	NM_003391	NP_003382	wingless-type MMTV integration site family member 2
WNT2B	NM_004185	NP_004176	wingless-type MMTV integration site family, member 2B
WNT3	NM_030753	NP_110380	wingless-type MMTV integration site family, member 3
WNT3A	NM_033131	NP_149122	wingless-type MMTV integration site family, member 3A
WNT4	NM_030761	NP_110388	wingless-type MMTV integration site family, member 4
WNT5A	NM_001256105	NP_001243034	wingless-type MMTV integration site family, member 5A
WNT5B	NM_030775	NP_110402	wingless-type MMTV integration site family, member 5B
WNT6	NM_006522	NP_006513	wingless-type MMTV integration site family, member 6
WNT7A	NM_004625	NP_004616	wingless-type MMTV integration site family, member 7A

(*Continued*)

Gene name	Nucleotide accession number	Protein accession number	Official full name
WNT7B	NM_058238	NP_478679	wingless-type MMTV integration site family, member 7B
WNT8A	NM_058244	NP_490645	wingless-type MMTV integration site family, member 8A
WNT8B	NM_003393	NP_003384	wingless-type MMTV integration site family, member 8B
WNT9A	NM_003395	NP_003386	wingless-type MMTV integration site family, member 9A
WNT9B	NM_003396	NP_003387	wingless-type MMTV integration site family, member 9B

Supplementary Table S7. Sorafenib-ADME candidate genes

Supplementary Table S8. Pathway analysis of single nuclear variations, small insertions, and deletions

Number of total KEGG orthologs[1]	Number of KEGG orthologs[1]	*p*-value	KEGG pathway name	Affected NCBI gene list
19	2	0.00012	Drug metabolism - cytochrome P450	FMO3, ALDH3B1
19	2	0.00012	Graft-versus-host disease	HLA-DQB1, HLA-DRB1, HLA-A, HLA-DRB5, HLA-B, HLA-DQA1
19	2	0.00012	Allograft rejection	HLA-DQB1, HLA-DRB1, HLA-A, HLA-DRB5, HLA-B, HLA-DQA1
22	2	0.00019	Autoimmune thyroid disease	HLA-DQB1, HLA-DRB1, HLA-A, HLA-DRB5, HLA-B, HLA-DQA1
22	2	0.00019	Type I diabetes mellitus	HLA-DQB1, HLA-DRB1, HLA-A, HLA-DRB5, HLA-B, HLA-DQA1
30	2	0.0005	Sphingolipid metabolism	SMPD1, SMPD2
34	2	0.00072	Intestinal immune network for IgA production	HLA-DQB1, HLA-DRB1, HLA-DRB5, MADCAM1, HLA-DQA1
36	2	0.00086	Viral myocarditis	HLA-DQB1, HLA-DRB1, HLA-A, HLA-DRB5, HLA-B, HLA-DQA1
36	2	0.00086	Glycolysis/ Gluconeogenesis	PGM1, ALDH3B1
86	3	0.00099	Cell adhesion molecules (CAMs)	HLA-DQB1, HLA-DRB1, HLA-A, HLA-DRB5, MADCAM1, HLA-B, HLA-DQA1

[1]Affected genes were assigned as Kyoto Encyclopedia of Genes and Genomes (KEGG) orthologs by using the KEGG genome annotation pipeline.

Supplementary Table S9. Genotype of 174 patients in validation sets for SNPs in SLC15A2

ID	rs Number			ID	rs Number		
	rs2257212	rs1143671	rs1143672		rs2257212	rs1143671	rs1143672
1	A/A	T/T	A/A	88	A/G	C/T	A/G
2	A/A	T/T	A/A	89	A/A	T/T	A/A
3	A/A	T/T	A/A	90	A/A	T/T	A/A
4	A/A	T/T	A/A	91	A/A	T/T	
5	A/G	C/T	A/G	92	A/A	T/T	A/A
6	A/G	C/T	A/G	93	A/A	T/T	A/A
7	A/A	T/T	A/A	94	A/G	C/T	A/G
8	A/A	T/T	A/A	95	A/A	T/T	A/A
9	A/A	T/T	A/A	96	A/A	T/T	A/A
10	A/A	T/T	A/A	97	G/G	C/C	G/G
11	A/A	T/T	A/A	98	A/G	C/T	A/G
12	A/G	C/T	A/G	99	A/A	T/T	A/A
13	A/G	C/T	A/G	100	A/A	T/T	A/A
14	A/A	T/T	A/A	101	G/G	C/C	G/G
15	A/A	T/T	A/A	102	A/G	C/T	A/G
16	A/G	C/T	A/G	103	A/A	T/T	A/A
17	A/G	C/T	A/G	104	A/G	C/T	A/G
18	A/G	C/T	A/G	105	A/A	T/T	A/A
19	A/A	T/T	A/A	106	A/G	C/T	A/G
20	A/G	C/T	A/G	107	A/A	T/T	A/A
21	A/A	T/T	A/A	108	A/A	T/T	A/A
22	A/A	T/T	A/A	109	A/G	C/T	A/G
23	A/A	T/T	A/A	110	A/A	T/T	A/A
24	A/G	C/T	A/G	111	A/G	C/T	
25	A/A	T/T	A/A	112	A/A	T/T	A/A
26	A/A	T/T	A/A	113	G/G	C/C	G/G
27	A/A	T/T	A/A	114	A/A	T/T	A/A
28	A/A	T/T	A/A	115	A/A	T/T	A/A
29	A/A	T/T	A/A	116	A/A	T/T	A/A
30	A/A	T/T	A/A	117	A/G	C/T	A/G
31	A/A	T/T	A/A	118	A/G	C/T	A/G
32	A/A	T/T	A/A	119	A/G	C/T	A/G
33	A/G	C/T	A/G	120	A/A	T/T	A/A
34	A/A	T/T	A/A	121	A/A	T/T	A/A
35	A/G	C/T	A/G	122	A/G	C/T	A/G
36	A/A	T/T	A/A	123	A/A	T/T	A/A

(*Continued*)

ID	rs Number			ID	rs Number		
	rs2257212	rs1143671	rs1143672		rs2257212	rs1143671	rs1143672
37	A/A	T/T	A/A	124	A/A	T/T	A/A
38	A/A	T/T	A/A	125	A/A	T/T	A/A
39	A/A	T/T	A/A	126	G/G	C/C	G/G
40	A/A	T/T	A/A	127	A/A	T/T	A/A
41	A/A	T/T	A/A	128	A/A	T/T	A/A
42	A/G	C/T	A/G	129	A/G	C/T	A/G
43	A/G	C/T	A/G	130	A/A	T/T	A/A
44	A/G	C/T	A/G	131	A/G	C/T	A/G
45	A/G	C/T	A/G	132	A/A	T/T	A/A
46	A/A	T/T	A/A	133	A/A	T/T	A/A
47	G/G	C/C	G/G	134	A/A	T/T	A/A
48	A/A	T/T	A/A	135	A/A	T/T	A/A
49	A/A	T/T	A/A	136	A/A	T/T	A/A
50	A/A	T/T	A/A	137	A/A	T/T	A/A
51	A/A	T/T	A/A	138	A/A	T/T	A/A
52	A/A	T/T	A/A	139	A/G	C/T	A/G
53	A/A	T/T	A/A	140	A/A	T/T	A/A
54	A/G	C/T	A/G	141	A/G	C/T	A/G
55	A/G	C/T	A/G	142	G/G	C/C	G/G
56	A/G	C/T	A/G	143	A/A	T/T	A/A
57	A/G	C/T	A/G	144	A/A	T/T	A/A
58	A/G	C/T	A/G	145	A/A	T/T	A/A
59	A/A	T/T	A/A	146	A/G	C/T	A/G
60	G/G	C/C	G/G	147	A/A	T/T	A/A
61	A/G	C/T	A/G	148	A/A	T/T	A/A
62	A/G	C/T	A/G	149	A/G	C/T	A/G
63	A/A	T/T	A/A	150	A/A	T/T	A/A
64	A/A	T/T	A/A	151	A/G	C/T	A/G
65	A/A	T/T	A/A	152	A/G	C/T	A/G
66	A/G	C/T	A/G	153	A/A	T/T	A/A
67	A/A	T/T	A/A	154	A/A	T/T	A/A
68	A/A	T/T	A/A	155	A/A	T/T	A/A
69	A/A	T/T	A/A	156	A/G	C/T	A/G
70	A/G	C/T	A/G	157	A/G	C/T	A/G
71	A/G	C/T	A/G	158	A/A	T/T	A/A
72	A/A	T/T	A/A	159	A/G	C/T	A/G

(*Continued*)

ID	rs Number			ID	rs Number		
	rs2257212	rs1143671	rs1143672		rs2257212	rs1143671	rs1143672
73	A/G	C/T	A/G	160	A/A	T/T	A/A
74	A/G	C/T	A/G	161	A/A	T/T	A/A
75	A/A	T/T	A/A	162	A/G	C/T	A/G
76	A/G	C/T	A/G	163	A/A	T/T	A/A
77	A/A	T/T	A/A	164	G/G	C/C	G/G
78	G/G	C/C	G/G	165	A/G	C/T	A/G
79	A/G	C/T	A/G	166	G/G	C/C	G/G
80	A/A	T/T	A/A	167	A/G	C/T	A/G
81	A/A	T/T	A/A	168	A/A	T/T	A/A
82	A/G	C/T	A/G	169	A/G	C/T	A/G
83	A/A	T/T	A/A	170	A/G	C/T	A/G
84	A/G	C/T	A/G	171	A/A	T/T	A/A
85	A/A	T/T	A/A	172	A/A	T/T	A/A
86	A/A	T/T	A/A	173	A/A	T/T	A/A
87	A/G	C/T	A/G	174	A/A	T/T	A/A

Supplementary Table S10. Prediction of post-translational modification for variants in SLC15A2

Protein Name	Predicted Phosphorylated Sites		
	Serin (S)	Threonine (T)	Tyrosine (Y)
NM_001145998.mut.cds. aminoacids (Good responder for sorafenib)	9	2	2
NM_001145998.wt.cds. aminoacids (Poor responder for sorafenib)	7	2	2

Supplementary Table S11. Sequences of the primer sets used for SNV validation

gene	chr#[a]	ref[b]	variant[c]	position[d]	primer sequence 1	primer sequence 2
FMO3	chr1	G	A	171076965	GATGTTACCACTGAAAGGGATGG	GAAGCGACCTTGTGAATAGATGC
CYP8B1	chr3	C	T	42918296	AAGAATGACTGTATGCCCTTCCA	AAGTGTATAGGCAAGCAGTTGGG
SLC15A2	chr3	C	T	121643803	AGGGAAATAGGGTCTTGGGTGTA	TCTTTTTCAAACTGGGCAAAGAC
SLC15A2	chr3	C	T	121647285	GCTGAGTCAAAAAGCATCGAGTT	ATTGTTTTCATTTCCCACCACTG
SLC15A2	chr3	G	A	121648167	TTACCAAGGATCTGCCTGATGAT	ATCTTCGAATCCCACATGAGAAA
UGT2B15	chr4	G	A	69526531	ATGGCGACACGTCTTCAAAATAG	GGGAGAAAGGGAGAAAAACAAAA
DDR1	chr6	A	C	30859354	AGATGGACTCCTGTCTTACACCG	GGGTGCCTTTTTCATACAGTGTC
DDR1	chr6	A	C	30865203	CTAGAGAGAACAATGGCAGAGCC	CACTGAGGAACTGGTTGAGGTC
ABCB1	chr7	G	C	87160617	ACAATGGCCTGAAAACTGAAAAA	CATTGCAATAGCAGGAGTTGTTG
PON3	chr7	G	A	95026159	TCCTACCTCAATTCCTCAGATGG	CCGTTTCCTGTCTTTTCCTTCTT
PDGFRL	chr8	C	T	17465536	AAGCAAAACGAAGATGTCAGAGG	CAAATCAGGATGAACTCCCAAAG
PDGFRL	chr8	G	A	17453555	AAACCTGGGAGTCCTCAACCTTA	AGGAACTGAGGTCCAGAGAGGAC
PDGFRL	chr8	C	T	17466211	CGTGCATTGGCACAATATATCAC	GACCACACACTGTCTTCTGTTGC
PDGFRL	chr8	A	G	17455052	TGACACTCACCTACAAAAGCAGG	TCCTTGCTAAAACACCACTGTGA
PDGFRL	chr8	A	G	17457428	ATGTCCTCCTTCCCTGATCTACC	TTATCAGAGAGGAAGATGGCTGC
PDGFRL	chr8	C	T	17465823	CTTTGGGAGTTCATCCTGATTTG	GTGATATATTGTGCCAATGCACG
PDGFRL	chr8		T	17455059	TGACACTCACCTACAAAAGCAGG	TCCTTGCTAAAACACCACTGTGA
PDGFRL	chr8	C	T	17452927	TCCAAGTTCCACTTGAGTTTTCC	GCTCTTGTTTGTTTAGGTCCAGG
PDGFRL	chr8	T	C	17466167	CGTGCATTGGCACAATATATCAC	TGTCTTCTGTTGCTCTGTCCTTG
MUSK	chr9	G	A	113538121	ACACAGAATTTAGGCTCTGCCAC	CCAAAGTCTTGGGAGAACTCTGT
ALDH3B1	chr11	G	A	67795298	TGAGGCTCAGAGGGGAGAAGTAG	ACAGCTGTCATGGTGGTCTACAG
ALDH3B1	chr11	G	A	67795352	TGAGGCTCAGAGGGGAGAAGTAG	ACAGCTGTCATGGTGGTCTACAG
FLT1	chr13	T	A	28894680	ACATGCTGTGTCAGCACCTTCTA	ACCAGTTTCTAGACCAGGGGTGT
ALDH6A1	chr14	T	A	74551517	GTGATTGGTTAGGAGCGAAAATG	CAAAGAGAAACCCTATCCCCAAC
ALDH6A1	chr14	C	A	74551525	GTGATTGGTTAGGAGCGAAAATG	CAAAGAGAAACCCTATCCCCAAC
ALDH6A1	chr14	C	T	74551975	CTTTCTTGGGCTCTTCTCCTTTC	GGTTTGTGAGAATCATTCCATCC

[a]chromosome, Chromosome on which the variation is located.
[b]ref, nucleotide at the same position in the Human reference genome sequence version 19/build 37.
[c]variant, nucleotide at the variantion site
[d]position, nucleotide position of the variant allele in the Human reference genome sequence version 19/build 37
