# Supplementary material for: SLC15A2 genomic variation is associated with the extraordinary response of sorafenib treatment: whole-genome analysis in patients with hepatocellular carcinoma
Source: Oncotarget. 2015 Apr 22;6(18):16449–60. doi: 10.18632/oncotarget.3758 (PMC4599281; doi:10.18632/oncotarget.3758)
Supplement: Supplementary file 2 [file oncotarget-06-16449-s002.doc]

Supplementary Table 7. Sorafenib-ADME candidate genes

| **Gene name** | **Nucleotide accession number** | **Protein accession number** | **Official full name** |
| --- | --- | --- | --- |
| ABCA1 | NM_005502 | NP_005493 | ATP-binding cassette, sub-family A (ABC1), member 1 |
| ABCA4 | NM_000350 | NP_000341 | ATP-binding cassette, sub-family A (ABC1), member 4 |
| ABCB1 | NM_000927 | NP_000918 | ATP-binding cassette, sub-family B (MDR/TAP), member 1 |
| ABCB11 | NM_003742 | NP_003733 | ATP-binding cassette, sub-family B (MDR/TAP), member 11 |
| ABCB4 | NM_000443 | NP_000434 | ATP-binding cassette, sub-family B (MDR/TAP), member 4 |
| ABCB5 | NM_029961 | NP_084237 | ATP-binding cassette, sub-family B (MDR/TAP), member 5 |
| ABCB6 | NM_005689 | NP_005680 | ATP-binding cassette, sub-family B (MDR/TAP), member 6 |
| ABCB7 | NM_004299 | NP_004290 | ATP-binding cassette, sub-family B (MDR/TAP), member 7 |
| ABCB8 | NM_007188 | NP_009119 | ATP-binding cassette, sub-family B (MDR/TAP), member 8 |
| ABCC1 | NM_004996 | NP_004987 | ATP-binding cassette, sub-family C (CFTR/MRP), member 1 |
| ABCC10 | NM_033450 | NP_258261 | ATP-binding cassette, sub-family C (CFTR/MRP), member 10 |
| ABCC11 | NM_032583 | NP_115972 | ATP-binding cassette, sub-family C (CFTR/MRP), member 11 |
| ABCC12 | NM_033226 | NP_150229 | ATP-binding cassette, sub-family C (CFTR/MRP), member 12 |
| ABCC13 | NM_001032930 | NP_001028102 | Putative ATP-binding cassette transporter sub-family C member 13 |
| ABCC2 | NM_000392 | NP_000383 | ATP-binding cassette, sub-family C (CFTR/MRP), member 2 |
| ABCC3 | NM_003786 | NP_003777 | ATP-binding cassette, sub-family C (CFTR/MRP), member 3 |
| ABCC4 | NM_005845 | NP_005836 | ATP-binding cassette, sub-family C (CFTR/MRP), member 4 |
| ABCC5 | NM_005688 | NP_005679 | ATP-binding cassette, sub-family C (CFTR/MRP), member 5 |
| ABCC6 | NM_001171 | NP_001162 | ATP-binding cassette, sub-family C (CFTR/MRP), member 6 |
| ABCC8 | NM_000352 | NP_000343 | ATP-binding cassette, sub-family C (CFTR/MRP), member 8 |
| ABCC9 | NM_005691 | NP_005682 | ATP-binding cassette, sub-family C (CFTR/MRP), member 9 |
| ABCG1 | NM_004915 | NP_004906 | ATP-binding cassette, sub-family G (WHITE), member 1 |
| ABCG2 | NM_004827 | NP_004818 | ATP-binding cassette, sub-family G (WHITE), member 2 |
| ADH1A | NM_000667 | NP_000658 | alcohol dehydrogenase 1A (class I), alpha polypeptide |
| ADH1B | NM_000668 | NP_000659 | alcohol dehydrogenase 1B (class I), beta polypeptide |
| ADH1C | NM_000669 | NP_000660 | alcohol dehydrogenase 1C (class I), gamma polypeptide |
| ADH4 | NM_000670 | NP_000661 | alcohol dehydrogenase 4 (class II), pi polypeptide |
| ADH5 | NM_000671 | NP_000662 | alcohol dehydrogenase 5 (class III), chi polypeptide |
| ADH6 | NM_000672 | NP_000663 | alcohol dehydrogenase 6 (class V) |
| ADH7 | NM_000673 | NP_000664 | alcohol dehydrogenase 7 (class IV), mu or sigma polypeptide |
| ADHFE1 | NM_144650 | NP_653251 | alcohol dehydrogenase, iron containing, 1 |
| AHR | NM_001621 | NP_001612 | aryl hydrocarbon receptor |
| ALDH1A1 | NM_000689 | NP_000680 | aldehyde dehydrogenase 1 family, member A1 |
| ALDH1A2 | NM_003888 | NP_003879 | aldehyde dehydrogenase 1 family, member A2 |
| ALDH1A3 | NM_000693 | NP_000684 | aldehyde dehydrogenase 1 family, member A3 |
| ALDH1B1 | NM_000692 | NP_000683 | aldehyde dehydrogenase 1 family, member B1 |
| ALDH2 | NM_000690 | NP_000681 | aldehyde dehydrogenase 2 family (mitochondrial) |
| ALDH3A1 | NM_000691 | NP_000682 | aldehyde dehydrogenase 3 family, member A1 |
| ALDH3A2 | NM_000382 | NP_000373 | aldehyde dehydrogenase 3 family, member A2 |
| ALDH3B1 | NM_000694 | NP_000685 | aldehyde dehydrogenase 3 family, member B1 |
| ALDH3B2 | NM_000695 | NP_000686 | aldehyde dehydrogenase 3 family, member B2 |
| ALDH4A1 | NM_003748 | NP_003739 | aldehyde dehydrogenase 4 family, member A1 |
| ALDH5A1 | NM_001080 | NP_001071 | aldehyde dehydrogenase 5 family, member A1 |
| ALDH6A1 | NM_005589 | NP_005580 | aldehyde dehydrogenase 6 family, member A1 |
| ALDH7A1 | NM_001182 | NP_001173 | aldehyde dehydrogenase 7 family, member A1 |
| ALDH8A1 | NM_022568 | NP_072090 | aldehyde dehydrogenase 8 family, member A1 |
| ALDH9A1 | NM_000696 | NP_000687 | aldehyde dehydrogenase 9 family, member A1 |
| AOX1 | NM_001159 | NP_001150 | aldehyde oxidase 1 |
| ARNT | NM_001668 | NP_001659 | aryl hydrocarbon receptor nuclear translocator |
| ARSA | NM_000487 | NP_000478 | arylsulfatase A |
| ATP7A | NM_000052 | NP_000043 | ATPase, Cu++ transporting, alpha polypeptide |
| ATP7B | NM_000053 | NP_000044 | ATPase, Cu++ transporting, beta polypeptide |
| CAT | NM_001752 | NP_001743 | catalase |
| CBR1 | NM_001757 | NP_001748 | carbonyl reductase 1 |
| CBR3 | NM_001236 | NP_001227 | carbonyl reductase 3 |
| CDA | NM_001785 | NP_001776 | cytidine deaminase |
| CES1 | NM_001266 | NP_001257 | carboxylesterase 1 (monocyte/macrophage serine esterase 1) |
| CES2 | NM_003869 | NP_003860 | carboxylesterase 2 (intestine, liver) |
| CFTR | NM_000492 | NP_000483 | cystic fibrosis transmembrane conductance regulator (ATP-binding cassette sub-family C, member 7) |
| CHST1 | NM_003654 | NP_003645 | carbohydrate (keratan sulfate Gal-6) sulfotransferase 1 |
| CHST10 | NM_004854 | NP_004845 | carbohydrate sulfotransferase 10 |
| CHST11 | NM_018413 | NP_060883 | carbohydrate (chondroitin 4) sulfotransferase 11 |
| CHST12 | NM_018641 | NP_061111 | carbohydrate (chondroitin 4) sulfotransferase 12 |
| CHST13 | NM_027928 | NP_082204 | carbohydrate (chondroitin 4) sulfotransferase 13 |
| CHST2 | NM_004267 | NP_004258 | carbohydrate (N-acetylglucosamine-6-O) sulfotransferase 2 |
| CHST3 | NM_004273 | NP_004264 | carbohydrate (chondroitin 6) sulfotransferase 3 |
| CHST4 | NM_005769 | NP_005760 | carbohydrate (N-acetylglucosamine 6-O) sulfotransferase 4 |
| CHST5 | NM_019950 | NP_064334 | carbohydrate (N-acetylglucosamine 6-O) sulfotransferase 5 |
| CHST6 | NM_021615 | NP_067628 | carbohydrate (N-acetylglucosamine 6-O) sulfotransferase 6 |
| CHST7 | NM_019886 | NP_063939 | carbohydrate (N-acetylglucosamine 6-O) sulfotransferase 7 |
| CHST8 | NM_022467 | NP_071912 | carbohydrate (N-acetylgalactosamine 4-0) sulfotransferase 8 |
| CHST9 | NM_031422 | NP_113610 | carbohydrate (N-acetylgalactosamine 4-0) sulfotransferase 9 |
| CYB5R3 | NM_000398 | NP_000389 | cytochrome b5 reductase 3 |
| CYP11A1 | NM_000781 | NP_000772 | cytochrome P450, family 11, subfamily A, polypeptide 1 |
| CYP11B1 | NM_000497 | NP_000488 | cytochrome P450, family 11, subfamily B, polypeptide 1 |
| CYP11B2 | NM_000498 | NP_000489 | cytochrome P450, family 11, subfamily B, polypeptide 2 |
| CYP17A1 | NM_000102 | NP_000093 | cytochrome P450, family 17, subfamily A, polypeptide 1 |
| CYP19A1 | NM_000103 | NP_000094 | cytochrome P450, family 19, subfamily A, polypeptide 1 |
| CYP1A1 | NM_000499 | NP_000490 | cytochrome P450, family 1, subfamily A, polypeptide 1 |
| CYP1A2 | NM_000761 | NP_000752 | cytochrome P450, family 1, subfamily A, polypeptide 2 |
| CYP1B1 | NM_000104 | NP_000095 | cytochrome P450, family 1, subfamily B, polypeptide 1 |
| CYP20A1 | NM_030013 | NP_084289 | cytochrome P450, family 20, subfamily A, polypeptide 1 |
| CYP21A2 | NM_000500 | NP_000491 | cytochrome P450, family 21, subfamily A, polypeptide 2 |
| CYP24A1 | NM_000782 | NP_000773 | cytochrome P450, family 24, subfamily A, polypeptide 1 |
| CYP26A1 | NM_000783 | NP_000774 | cytochrome P450, family 26, subfamily A, polypeptide 1 |
| CYP26C1 | NM_183374 | NP_899230 | cytochrome P450, family 26, subfamily C, polypeptide 1 |
| CYP27A1 | NM_000784 | NP_000775 | cytochrome P450, family 27, subfamily A, polypeptide 1 |
| CYP27B1 | NM_000785 | NP_000776 | cytochrome P450, family 27, subfamily B, polypeptide 1 |
| CYP2A13 | NM_000766 | NP_000757 | cytochrome P450, family 2, subfamily A, polypeptide 13 |
| CYP2A6 | NM_000762 | NP_000753 | cytochrome P450, family 2, subfamily A, polypeptide 6 |
| CYP2A7 | NM_000764 | NP_000755 | cytochrome P450, family 2, subfamily A, polypeptide 7 |
| CYP2B6 | NM_000767 | NP_000758 | cytochrome P450, family 2, subfamily B, polypeptide 6 |
| CYP2C18 | NM_000772 | NP_000763 | cytochrome P450, family 2, subfamily C, polypeptide 18 |
| CYP2C19 | NM_000769 | NP_000760 | cytochrome P450, family 2, subfamily C, polypeptide 19 |
| CYP2C8 | NM_000770 | NP_000761 | cytochrome P450, family 2, subfamily C, polypeptide 8 |
| CYP2C9 | NM_000771 | NP_000762 | cytochrome P450, family 2, subfamily C, polypeptide 9 |
| CYP2D6 | NM_000106 | NP_000097 | cytochrome P450, family 2, subfamily D, polypeptide 6 |
| CYP2E1 | NM_000773 | NP_000764 | cytochrome P450, family 2, subfamily E, polypeptide 1 |
| CYP2F1 | NM_000774 | NP_000765 | cytochrome P450, family 2, subfamily F, polypeptide 1 |
| CYP2J2 | NM_000775 | NP_000766 | cytochrome P450, family 2, subfamily J, polypeptide 2 |
| CYP2R1 | NM_024514 | NP_078790 | cytochrome P450, family 2, subfamily R, polypeptide 1 |
| CYP2S1 | NM_028775 | NP_083051 | cytochrome P450, family 2, subfamily S, polypeptide 1 |
| CYP39A1 | NM_016593 | NP_057677 | cytochrome P450, family 39, subfamily A, polypeptide 1 |
| CYP3A4 | NM_017460 | NP_059488 | cytochrome P450, family 3, subfamily A, polypeptide 4 |
| CYP3A43 | NM_022820 | NP_073731 | cytochrome P450, family 3, subfamily A, polypeptide 43 |
| CYP3A5 | NM_000777 | NP_000768 | cytochrome P450, family 3, subfamily A, polypeptide 5 |
| CYP3A7 | NM_000765 | NP_000756 | cytochrome P450, family 3, subfamily A, polypeptide 7 |
| CYP46A1 | NM_006668 | NP_006659 | cytochrome P450, family 46, subfamily A, polypeptide 1 |
| CYP4A11 | NM_000778 | NP_000769 | cytochrome P450, family 4, subfamily A, polypeptide 11 |
| CYP4B1 | NM_000779 | NP_000770 | cytochrome P450, family 4, subfamily B, polypeptide 1 |
| CYP4F11 | NM_021187 | NP_067010 | cytochrome P450, family 4, subfamily F, polypeptide 11 |
| CYP4F12 | NM_023944 | NP_076433 | cytochrome P450, family 4, subfamily F, polypeptide 12 |
| CYP4F2 | NM_001082 | NP_001073 | cytochrome P450, family 4, subfamily F, polypeptide 2 |
| CYP4F3 | NM_000896 | NP_000887 | cytochrome P450, family 4, subfamily F, polypeptide 3 |
| CYP4F8 | NM_007253 | NP_009184 | cytochrome P450, family 4, subfamily F, polypeptide 8 |
| CYP4Z1 | NM_178134 | NP_835235 | cytochrome P450, family 4, subfamily Z, polypeptide 1 |
| CYP51A1 | NM_000786 | NP_000777 | cytochrome P450, family 51, subfamily A, polypeptide 1 |
| CYP7A1 | NM_000780 | NP_000771 | cytochrome P450, family 7, subfamily A, polypeptide 1 |
| CYP7B1 | NM_004820 | NP_004811 | cytochrome P450, family 7, subfamily B, polypeptide 1 |
| CYP8B1 | NM_004391 | NP_004382 | cytochrome P450, family 8, subfamily B, polypeptide 1 |
| DDO | NM_003649 | NP_003640 | D-aspartate oxidase |
| DHRS1 | NM_026819 | NP_081095 | dehydrogenase/reductase (SDR family) member 1 |
| DHRS12 | NM_024705 | NP_078981 | dehydrogenase/reductase (SDR family) member 12 |
| DHRS13 | NM_144683 | NP_653284 | dehydrogenase/reductase (SDR family) member 13 |
| DHRS2 | NM_005794 | NP_005785 | dehydrogenase/reductase (SDR family) member 2 |
| DHRS3 | NM_004753 | NP_004744 | dehydrogenase/reductase (SDR family) member 3 |
| DHRS4 | NM_021004 | NP_066284 | dehydrogenase/reductase (SDR family) member 4 |
| DHRS4L1 | NM_001082488 | NP_001075957 | dehydrogenase/reductase (SDR family) member 4 like 1 |
| DHRS4L2 | NM_198083 | NP_932349 | dehydrogenase/reductase (SDR family) member 4 like 2 |
| DHRS7 | NM_016029 | NP_057113 | dehydrogenase/reductase (SDR family) member 7 |
| DHRS7B | NM_015510 | NP_056325 | dehydrogenase/reductase (SDR family) member 7B |
| DHRS7C | NM_001013013 | NP_001013031 | dehydrogenase/reductase (SDR family) member 7C |
| DHRS9 | NM_005771 | NP_005762 | dehydrogenase/reductase (SDR family) member 9 |
| DHRSX | NM_145177 | NP_660160 | dehydrogenase/reductase (SDR family) X-linked |
| DPEP1 | NM_004413 | NP_004404 | dipeptidase 1 (renal) |
| DPYD | NM_000110 | NP_000101 | dihydropyrimidine dehydrogenase |
| EPHX1 | NM_000120 | NP_000111 | epoxide hydrolase 1, microsomal (xenobiotic) |
| EPHX2 | NM_001979 | NP_001970 | epoxide hydrolase 2, cytoplasmic |
| FMO1 | NM_002021 | NP_002012 | flavin containing monooxygenase 1 |
| FMO2 | NM_001460 | NP_001451 | flavin containing monooxygenase 2 (non-functional) |
| FMO3 | NM_006894 | NP_008825 | flavin containing monooxygenase 3 |
| FMO4 | NM_002022 | NP_002013 | flavin containing monooxygenase 4 |
| FMO5 | NM_001461 | NP_001452 | flavin containing monooxygenase 5 |
| FMO6P | NM_204579 | NP_989910 | flavin containing monooxygenase 6 pseudogene |
| GPX1 | NM_000581 | NP_000572 | glutathione peroxidase 1 |
| GPX2 | NM_002083 | NP_002074 | glutathione peroxidase 2 (gastrointestinal) |
| GPX3 | NM_002084 | NP_002075 | glutathione peroxidase 3 (plasma) |
| GPX4 | NM_002085 | NP_002076 | glutathione peroxidase 4 (phospholipid hydroperoxidase) |
| GPX5 | NM_001509 | NP_001500 | glutathione peroxidase 5 (epididymal androgen-related protein) |
| GPX6 | NM_117229 | NP_192897 | glutathione peroxidase 6 (olfactory) |
| GPX7 | NM_015696 | NP_056511 | glutathione peroxidase 7 |
| GSR | NM_000637 | NP_000628 | glutathione reductase |
| GSS | NM_000178 | NP_000169 | glutathione synthetase |
| GSTA1 | NM_008181 | NP_032207 | glutathione S-transferase alpha 1 |
| GSTA2 | NM_000846 | NP_000837 | glutathione S-transferase alpha 2 |
| GSTA3 | NM_000847 | NP_000838 | glutathione S-transferase alpha 3 |
| GSTA4 | NM_001512 | NP_001503 | glutathione S-transferase alpha 4 |
| GSTA5 | NM_153699 | NP_714543 | glutathione S-transferase alpha 5 |
| GSTCD | NM_024751 | NP_079027 | glutathione S-transferase, C-terminal domain containing |
| GSTK1 | NM_015917 | NP_057001 | glutathione S-transferase kappa 1 |
| GSTM1 | NM_000561 | NP_000552 | glutathione S-transferase mu 1 |
| GSTM2 | NM_000848 | NP_000839 | glutathione S-transferase mu 2 (muscle) |
| GSTM3 | NM_000849 | NP_000840 | glutathione S-transferase mu 3 (brain) |
| GSTM4 | NM_000850 | NP_000841 | glutathione S-transferase mu 4 |
| GSTM5 | NM_000851 | NP_000842 | glutathione S-transferase mu 5 |
| GSTO1 | NM_004832 | NP_004823 | glutathione S-transferase omega 1 |
| GSTO2 | NM_026619 | NP_080895 | glutathione S-transferase omega 2 |
| GSTP1 | NM_000852 | NP_000843 | glutathione S-transferase pi 1 |
| GSTT1 | NM_000853 | NP_000844 | glutathione S-transferase theta 1 |
| GSTT2 | NM_000854 | NP_000845 | glutathione S-transferase theta 2 |
| GSTZ1 | NM_001513 | NP_001504 | glutathione transferase zeta 1 |
| HAGH | NM_005326 | NP_005317 | hydroxyacylglutathione hydrolase |
| HNF4A | NM_000457 | NP_000448 | hepatocyte nuclear factor 4, alpha |
| HNMT | NM_006895 | NP_008826 | histamine N-methyltransferase |
| HSD11B1 | NM_005525 | NP_005516 | hydroxysteroid (11-beta) dehydrogenase 1 |
| HSD17B11 | NM_016245 | NP_057329 | hydroxysteroid (17-beta) dehydrogenase 11 |
| HSD17B14 | NM_016246 | NP_057330 | hydroxysteroid (17-beta) dehydrogenase 14 |
| IAPP | NM_000415 | NP_000406 | islet amyloid polypeptide |
| KCNJ11 | NM_000525 | NP_000516 | potassium inwardly-rectifying channel, subfamily J, member 11 |
| MAT1A | NM_000429 | NP_000420 | methionine adenosyltransferase I, alpha |
| METAP1 | NM_015143 | NP_055958 | methionyl aminopeptidase 1 |
| MGST1 | NM_019946 | NP_064330 | microsomal glutathione S-transferase 1 |
| MGST2 | NM_002413 | NP_002404 | microsomal glutathione S-transferase 2 |
| MGST3 | NM_004528 | NP_004519 | microsomal glutathione S-transferase 3 |
| MPO | NM_000250 | NP_000241 | myeloperoxidase |
| NAT1 | NM_000662 | NP_000653 | N-acetyltransferase 1 (arylamine N-acetyltransferase) |
| NAT2 | NM_000015 | NP_000006 | N-acetyltransferase 2 (arylamine N-acetyltransferase) |
| NNMT | NM_006169 | NP_006160 | nicotinamide N-methyltransferase |
| NOS1 | NM_000620 | NP_000611 | nitric oxide synthase 1 (neuronal) |
| NOS2A | NM_001104937 | NP_001098407 | nitric oxide synthase 2, inducible |
| NOS3 | NM_000603 | NP_000594 | nitric oxide synthase 3 (endothelial cell) |
| NR1I2 | NM_003889 | NP_003880 | nuclear receptor subfamily 1, group I, member 2 |
| NR1I3 | NM_005122 | NP_005113 | nuclear receptor subfamily 1, group I, member 3 |
| PDE3A | NM_000921 | NP_000912 | phosphodiesterase 3A, cGMP-inhibited |
| PDE3B | NM_000922 | NP_000913 | phosphodiesterase 3B, cGMP-inhibited |
| PLGLB1 | NM_001032392 | NP_001027564 | plasminogen-like B1 |
| PNMT | NM_002686 | NP_002677 | phenylethanolamine N-methyltransferase |
| PON1 | NM_000446 | NP_000437 | paraoxonase 1 |
| PON2 | NM_000305 | NP_000296 | paraoxonase 2 |
| PON3 | NM_000940 | NP_000931 | paraoxonase 3 |
| POR | NM_000941 | NP_000932 | P450 (cytochrome) oxidoreductase |
| PPARA | NM_005036 | NP_005027 | peroxisome proliferator-activated receptor alpha |
| PPARD | NM_006238 | NP_006229 | peroxisome proliferator-activated receptor delta |
| PPARG | NM_005037 | NP_005028 | peroxisome proliferator-activated receptor gamma |
| RXRA | NM_002957 | NP_002948 | retinoid X receptor, alpha |
| SERPINA7 | NM_000354 | NP_000345 | serpin peptidase inhibitor, clade A (alpha-1 antiproteinase, antitrypsin), member 7 |
| SLC10A1 | NM_003049 | NP_003040 | solute carrier family 10 (sodium/bile acid cotransporter family), member 1 |
| SLC10A2 | NM_000452 | NP_000443 | solute carrier family 10 (sodium/bile acid cotransporter family), member 2 |
| SLC13A1 | NM_019481 | NP_062354 | solute carrier family 13 (sodium/sulfate symporters), member 1 |
| SLC13A2 | NM_003984 | NP_003975 | solute carrier family 13 (sodium-dependent dicarboxylate transporter), member 2 |
| SLC13A3 | NM_022829 | NP_073740 | solute carrier family 13 (sodium-dependent dicarboxylate transporter), member 3 |
| SLC15A1 | NM_005073 | NP_005064 | solute carrier family 15 (oligopeptide transporter), member 1 |
| SLC15A2 | NM_021082 | NP_066568 | solute carrier family 15 (H+/peptide transporter), member 2 |
| SLC16A1 | NM_003051 | NP_003042 | solute carrier family 16, member 1 (monocarboxylic acid transporter 1) |
| SLC19A1 | NM_017299 | NP_058995 | solute carrier family 19 (folate transporter), member 1 |
| SLC22A1 | NM_003057 | NP_003048 | solute carrier family 22 (organic cation transporter), member 1 |
| SLC22A10 | NM_001039752 | NP_001034841 | solute carrier family 22, member 10 |
| SLC22A11 | NM_018484 | NP_060954 | solute carrier family 22 (organic anion/urate transporter), member 11 |
| SLC22A12 | NM_009203 | NP_033229 | solute carrier family 22 (organic anion/urate transporter), member 12 |
| SLC22A13 | NM_004256 | NP_004247 | solute carrier family 22 (organic anion transporter), member 13 |
| SLC22A14 | NM_004803 | NP_004794 | solute carrier family 22, member 14 |
| SLC22A15 | NM_018420 | NP_060890 | solute carrier family 22, member 15 |
| SLC22A16 | NM_027572 | NP_081848 | solute carrier family 22 (organic cation/carnitine transporter), member 16 |
| SLC22A17 | NM_016609 | NP_057693 | solute carrier family 22, member 17 |
| SLC22A18 | NM_002555 | NP_002546 | solute carrier family 22, member 18 |
| SLC22A18AS | NM_007105 | NP_009036 | solute carrier family 22 (organic cation transporter), member 18 antisense |
| SLC22A2 | NM_003058 | NP_003049 | solute carrier family 22 (organic cation transporter), member 2 |
| SLC22A3 | NM_011395 | NP_035525 | solute carrier family 22 (extraneuronal monoamine transporter), member 3 |
| SLC22A4 | NM_003059 | NP_003050 | solute carrier family 22 (organic cation/ergothioneine transporter), member 4 |
| SLC22A5 | NM_003060 | NP_003051 | solute carrier family 22 (organic cation/carnitine transporter), member 5 |
| SLC22A6 | NM_004790 | NP_004781 | solute carrier family 22 (organic anion transporter), member 6 |
| SLC22A7 | NM_006672 | NP_006663 | solute carrier family 22 (organic anion transporter), member 7 |
| SLC22A8 | NM_004254 | NP_004245 | solute carrier family 22 (organic anion transporter), member 8 |
| SLC22A9 | NM_080866 | NP_543142 | solute carrier family 22 (organic anion transporter), member 9 |
| SLC27A1 | NM_011977 | NP_036107 | solute carrier family 27 (fatty acid transporter), member 1 |
| SLC28A1 | NM_004213 | NP_004204 | solute carrier family 28 (sodium-coupled nucleoside transporter), member 1 |
| SLC28A2 | NM_004212 | NP_004203 | solute carrier family 28 (sodium-coupled nucleoside transporter), member 2 |
| SLC28A3 | NM_022127 | NP_071410 | solute carrier family 28 (sodium-coupled nucleoside transporter), member 3 |
| SLC29A1 | NM_004955 | NP_004946 | solute carrier family 29 (nucleoside transporters), member 1 |
| SLC29A2 | NM_001532 | NP_001523 | solute carrier family 29 (nucleoside transporters), member 2 |
| SLC2A4 | NM_001042 | NP_001033 | solute carrier family 2 (facilitated glucose transporter), member 4 |
| SLC2A5 | NM_003039 | NP_003030 | solute carrier family 2 (facilitated glucose/fructose transporter), member 5 |
| SLC5A6 | NM_021095 | NP_066918 | solute carrier family 5 (sodium-dependent vitamin transporter), member 6 |
| SLC6A6 | NM_003043 | NP_003034 | solute carrier family 6 (neurotransmitter transporter, taurine), member 6 |
| SLC7A5 | NM_003486 | NP_003477 | solute carrier family 7 (cationic amino acid transporter, y+ system), member 5 |
| SLC7A7 | NM_011405 | NP_035535 | solute carrier family 7 (cationic amino acid transporter, y+ system), member 7 |
| SLC7A8 | NM_012244 | NP_036376 | solute carrier family 7 (amino acid transporter, L-type), member 8 |
| SLCO1A2 | NM_021094 | NP_066580 | solute carrier organic anion transporter family, member 1A2 |
| SLCO1B1 | NM_006446 | NP_006437 | solute carrier organic anion transporter family, member 1B1 |
| SLCO1B3 | NM_019844 | NP_062818 | solute carrier organic anion transporter family, member 1B3 |
| SLCO1C1 | NM_017435 | NP_059131 | solute carrier organic anion transporter family, member 1C1 |
| SLCO2A1 | NM_005630 | NP_005621 | solute carrier organic anion transporter family, member 2A1 |
| SLCO2B1 | NM_007256 | NP_009187 | solute carrier organic anion transporter family, member 2B1 |
| SLCO3A1 | NM_013272 | NP_037404 | solute carrier organic anion transporter family, member 3A1 |
| SLCO4A1 | NM_016354 | NP_057438 | solute carrier organic anion transporter family, member 4A1 |
| SLCO4C1 | NM_172658 | NP_766246 | solute carrier organic anion transporter family, member 4C1 |
| SLCO5A1 | NM_030958 | NP_112220 | solute carrier organic anion transporter family, member 5A1 |
| SLCO6A1 | NM_173488 | NP_775759 | solute carrier organic anion transporter family, member 6A1 |
| SOD1 | NM_000454 | NP_000445 | superoxide dismutase 1, soluble |
| SOD2 | NM_000636 | NP_000627 | superoxide dismutase 2, mitochondrial |
| SOD3 | NM_003102 | NP_003093 | superoxide dismutase 3, extracellular |
| SULF1 | NM_015170 | NP_055985 | sulfatase 1 |
| SULT1A1 | NM_001055 | NP_001046 | sulfotransferase family, cytosolic, 1A, phenol-preferring, member 1 |
| SULT1A2 | NM_001054 | NP_001045 | sulfotransferase family, cytosolic, 1A, phenol-preferring, member 2 |
| SULT1A3 | NM_177552 | NP_808220 | sulfotransferase family, cytosolic, 1A, phenol-preferring, member 3 |
| SULT1B1 | NM_014465 | NP_055280 | sulfotransferase family, cytosolic, 1B, member 1 |
| SULT1C1 | NM_018751 | NP_061221 | sulfotransferase family, cytosolic, 1C, member 1 |
| SULT1C2 | NM_001056 | NP_001047 | sulfotransferase family, cytosolic, 1C, member 2 |
| SULT1E1 | NM_005420 | NP_005411 | sulfotransferase family 1E, estrogen-preferring, member 1 |
| SULT2A1 | NM_003167 | NP_003158 | sulfotransferase family, cytosolic, 2A, dehydroepiandrosterone (DHEA)-preferring, member 1 |
| SULT2B1 | NM_004605 | NP_004596 | sulfotransferase family, cytosolic, 2B, member 1 |
| SULT4A1 | NM_013873 | NP_038901 | sulfotransferase family 4A, member 1 |
| TAP1 | NM_000593 | NP_000584 | transporter 1, ATP-binding cassette, sub-family B (MDR/TAP) |
| TAP2 | NM_000544 | NP_000535 | transporter 2, ATP-binding cassette, sub-family B (MDR/TAP) |
| TPMT | NM_000367 | NP_000358 | thiopurine S-methyltransferase |
| UGT1A1 | NM_000463 | NP_000454 | UDP glucuronosyltransferase 1 family, polypeptide A1 |
| UGT1A10 | NM_019075 | NP_061948 | UDP glucuronosyltransferase 1 family, polypeptide A10 |
| UGT1A3 | NM_019093 | NP_061966 | UDP glucuronosyltransferase 1 family, polypeptide A3 |
| UGT1A4 | NM_007120 | NP_009051 | UDP glucuronosyltransferase 1 family, polypeptide A4 |
| UGT1A5 | NM_019078 | NP_061951 | UDP glucuronosyltransferase 1 family, polypeptide A5 |
| UGT1A6 | NM_001072 | NP_001063 | UDP glucuronosyltransferase 1 family, polypeptide A6 |
| UGT1A7 | NM_019077 | NP_061950 | UDP glucuronosyltransferase 1 family, polypeptide A7 |
| UGT1A8 | NM_019076 | NP_061949 | UDP glucuronosyltransferase 1 family, polypeptide A8 |
| UGT1A9 | NM_021027 | NP_066307 | UDP glucuronosyltransferase 1 family, polypeptide A9 |
| UGT2A1 | NM_006798 | NP_006789 | UDP glucuronosyltransferase 2 family, polypeptide A1 |
| UGT2B10 | NM_001075 | NP_001066 | UDP glucuronosyltransferase 2 family, polypeptide B10 |
| UGT2B11 | NM_001073 | NP_001064 | UDP glucuronosyltransferase 2 family, polypeptide B11 |
| UGT2B15 | NM_001076 | NP_001067 | UDP glucuronosyltransferase 2 family, polypeptide B15 |
| UGT2B17 | NM_001077 | NP_001068 | UDP glucuronosyltransferase 2 family, polypeptide B17 |
| UGT2B28 | NM_053039 | NP_444267 | UDP glucuronosyltransferase 2 family, polypeptide B28 |
| UGT2B4 | NM_021139 | NP_066962 | UDP glucuronosyltransferase 2 family, polypeptide B4 |
| UGT2B7 | NM_001074 | NP_001065 | UDP glucuronosyltransferase 2 family, polypeptide B7 |
| UGT8 | NM_003360 | NP_003351 | UDP glycosyltransferase 8 |
| XDH | NM_000379 | NP_000370 | xanthine dehydrogenase |
